# Vitamin B_12_ Levels of Subjects Aged 0-24 Year(s) in Konya, Turkey

**Published:** 2014-12

**Authors:** Fatih Akin, Haluk Yavuz, Said Bodur, Aysel Kiyici

**Affiliations:** ^1^Konya Training and Research Hospital, Department of Pediatrics, Konya, Turkey; ^2^Department of Pediatrics, Faculty of Medicine, Selcuk University, Konya, Turkey; ^3^Department of Public Health, Faculty of Medicine, Selcuk University, Konya, Turkey; ^4^Department of Biochemistry, Faculty of Medicine, Selcuk University, Konya, Turkey

**Keywords:** Childhood, Nutrition, Reference ranges, Vitamin B_12_, Turkey

## Abstract

Research reports indicate that vitamin B_12_ levels show racial differences, which suggests that using the reference ranges of varied populations may lead to inaccurate results. This study aimed to determine normal serum levels of vitamin B_12_ among children and young people in the Konya region of Turkey. It evaluated 1,109 samples; 54 were from cord-blood and 1,055 were from healthy subjects aged 0-24 year(s), who were admitted to primary healthcare centres. The normal reference levels obtained for vitamin B_12_ at 2.5-97.5 percentile (P_2.5_-P_97.5_) range were 127-606 pg/mL for girls, 127-576 pg/mL for boys, and 127-590 pg/mL for the entire study group. The reported reference values for vitamin B_12_ in other studies were higher than the current results. Vitamin B_12_ levels vary from country to country; comparisons between countries may not be valid, and normal levels for each population should be obtained.

## INTRODUCTION

Vitamin B_12_ is a water-soluble vitamin that is an essential co-factor in some biochemical reactions and required for the synthesis of both RNA and DNA. Its deficiency may cause disorders, especially in the haematologic, neurologic, and gastrointestinal systems. Deficiency in infancy may lead to mental retardation and may have lifelong effects (1).

Reports indicate that vitamin B_12_ levels show racial differences; thus, using the reference ranges of varied populations may lead to inaccurate results (2-6). Therefore, normal levels that are valid for each population should be obtained. To the authors’ knowledge, no research in the literature addressed reference ranges of vitamin B_12_ for the population of Konya region of Turkey. The authors designed this work to determine the normal levels of vitamin B_12_ in this region.

## MATERIALS AND METHODS

The study was conducted during May 2006 to March 2007. It screened samples of 1,109 subjects aged 0-24 year(s) (560 boys, 549 girls), including 54 cord-blood samples. The cord-blood was obtained through the umbilical cord at the time of birth in the Department of Obstetrics and Gynaecology, Selcuk University Hospital in Turkey. The other subjects were patients admitted to primary healthcare centres for any complaint. The study followed the guidelines set forth in the Declaration of Helsinki, and the procedures were approved by the Ethics Committee of Selcuk University Meram Medical Faculty.

Blood samples were obtained from persons undergoing blood analyses for other reasons. Twenty-eight of the cord-blood samples were collected from male babies and 26 from females. The distribution of subjects by age was as follows: newborn group (45 subjects; 23 boys, 22 girls), 1-12 month(s) (38 subjects; 17 boys, 21 girls), and 13-24 months (52 subjects; 32 boys, 20 girls). In each age-group from 24 months to 24 years, there were 20 boys and 20 girls for every year. Samples were obtained after administering a questionnaire consisting of 16 items about conditions that can affect the status of vitamin B_12_ to determine suitability of subjects for the study.

The first section of the questionnaire recorded age, sex, height, weight, body mass index [BMI: weight (kg)/height (m)^2^] as well as occupation, educational status, and monthly income of parents. The second section queried the medical history (disease, operations, drugs), and the third section established the nutritional status of the study group, with questions regarding fish, chicken, or red meat consumption in the previous three days and the frequency of seafood, red meat and offal consumption. Since the most important source of vitamin B_12_ is red meat, consumption of red meat at least twice per week was the standard for inclusion into the study, and the subjects consuming less than that were excluded. The third section of the questionnaire asked also about the foods normally consumed at breakfast. In children who were breastfed, the study considered the nutritional status of the mother. When essential, the results of a physical examination informed the decision of including a patient. As the study was designed to determine the normal levels of vitamin B_12_, those considered to have nutritional deficiency were not included. Other exclusion criteria were: (i) history of renal, haematologic, gastrointestinal or metabolic disease (leukaemia, polycythaemia, hypereosinophilia, cystic fibrosis, hepatitis, cirrhosis, cancer), gastrointestinal surgery, malnutrition, growth retardation, malabsorption, or prematurity; (ii) taking drugs containing vitamin B_12_; (iii) pregnant women taking B_12_-containing vitamins (for cord-blood); (iv) infants being formula-fed; (v) use of any anti-epileptic drug; (vi) drinking alcoholic beverages and/or smoking; (vii) history of parasitic infections; and (viii) emigrants of other races.

Researchers obtained informed consent from all participants or their parents (for children below 18 years of age). The subjects included in the study were divided into three groups according to the monthly family income [<600 Turkish Liras (TL)], 600-1,299 TL, and >1,200 TL; 1 US$ equalled 1.4 TL at the time of the study. The study divided the educational status of parents into five groups (non-literate, primary school education, secondary school education, high school education, and a university degree).

The study did not investigate the exact presence of conditions that affect vitamin B_12_ levels as this requires numerous analyses that could have rendered the study impracticable in terms of time and financial cost. As in many similar studies, the declarations of subjects and parents were taken into account (7-9).

As vitamin B_12_ levels are not affected by daily nutrition and do not vary throughout the day, samples were obtained between 08:00 am and 16:00 pm (10). Sera were separated by centrifugation and stored at −20 ^o^C until the day of assay. The Biochemistry Laboratory of Selcuk University Meram Medical Faculty measured the levels of vitamin B_12_ with original Beckman kits (Beckman Coulter, CA, USA) by the chemiluminescent method on UniCel DXI 800 Access immunoassay device (Beckman Coulter, CA, USA). The recommended reference interval by the manufacturer was 180-914 pg/mL. The intra-assay precision values of the kit were 5% and 11.4% for 88 pg/mL and 914 pg/mL respectively. The inter-assay precision values were 8.5% and 11.4% respectively. The analytic sensitivity of the kits was 50 pg/mL and analytic specificity was 99.5%.

### Statistical analysis

Statistical analyses were performed using the Statistical Package for the Social Sciences (SPSS) (version 13.0). Data were summarized as mean±standard deviation and median. While Mann-Whitney U-test was used in comparing two groups, one-way variance analysis was used when more than two groups were compared. Association of parameters was determined by Spearman's correlation coefficient. A value of p<0.05 was considered to be statistically significant.

## RESULTS

### Demography

Evaluations of parental educational status found that the percentage of mothers who were non-literate, had primary-, secondary-, or high school-level education, or had a university degree were 4.4%, 67%, 8.5%, 13.1%, and 6.7% respectively. These values for the fathers were 0.8%, 47.4%, 13.2%, 22.2%, and 16.4% respectively. Most of the mothers (90%) were housewife, and 55.7% of the fathers were tradesmen. Monthly incomes among families were 600 TL in 22.1% and 600-1,200 TL in 48.5%.

An evaluation of major sources for intake of vitamin B_12_ showed that intake rates of fish and offal were low. Cheese, yogurt and egg intakes were high at breakfast. Consumption frequency of red meat was at least two times per week for the whole study group (Table 1).

### Vitamin B_12_ levels

Mean vitamin B_12_ levels in boys, girls, and the general population (aged 1 month to 24 years) were 262.7±130 pg/mL, 263±124 pg/mL, and 263±127 pg/mL respectively. Table 2 gives the vitamin B_12_ levels by age-group; Table 3 shows the results for cord-blood and newborns. Evaluation of these results revealed that vitamin B_12_ levels of both male and female subjects in the 1-5 year(s) old and the 6-11 years old groups were significantly higher than levels among the newborns (p<0.05 vs p<0.01). The levels in 1-5 year(s) old males and both 1-5 year(s) old and 6-11 years old females were significantly higher than the levels found in the 12-17 years old subjects (p<0.05). Additionally, the levels in the 1-5 year(s) old and the 6-12 years old females were significantly higher than the levels in 18-24 years old subjects (p<0.01).

**Table 1.** Nutritional status of the study group

Food intake pattern	Number	Percentage (%)
Frequency of meat consumption		
Several times per week (>2/week) Almost every day	1,057 52	95.3 4.6
Meat consumption in the last 3 days		
No Yes Red meat Fish Poultry	174 935 538 177 220	15.6 84.3 48.5 15.9 19.8
Frequency of fish consumption		
Never Rarely Once per month Once or more per week	165 338 344 262	6.7 30.4 31 23.6
Frequency of offal consumption		
Never Rarely Several times per month	752 268 89	67.8 24.1 8
Foods consumed at breakfast		
No breakfast Jam/Honey Butter Cheese-yoghurt-egg Olive Mother's milk Mother's milk+supplements Others	24 60 45 613 152 59 52 104	2.1 5.4 4 55.2 13.7 5.3 4.6 9.3
Drinks at breakfast		
Nothing Tea Milk Fruit juice/Others	31 783 239 56	2.7 70.6 21.5 5

Evaluation of the subjects according to gender revealed that vitamin B_12_ levels were significantly higher in females at the age of 7 and 14 years and in males at the age of 21 years when compared with the opposite gender (p<0.05). Considering correlations of vitamin B_12_ levels with age, height, weight, and BMI, a negative association was obtained with each (p<0.001).

Associations of educational status with vitamin B_12_ levels in fathers revealed that children of fathers who had high school education had significantly higher levels than children of fathers who were non-literate or who had a primary school education (286.6±147.5 pg/mL vs 248.2±133.7 pg/mL and 255.8±140.5 pg/mL respectively, p<0.05) while children of mothers with a university degree had significantly higher vitamin B_12_ levels than children of non-literate mothers (316.4±160.0 pg/mL vs 275.1±197.6 pg/mL respectively, p<0.05). Vitamin B_12_ levels showed no significant differences for the parents’ occupations or monthly incomes.

### Dietary habits

Assessment of data showed no statistically significant difference for the frequency of red meat consumption. A significant difference did exist between subjects who never consumed fish and those who consumed it once or more per month (238.7±111.9 pg/mL vs 270.4±144.7 pg/mL and 285.0±151.4 pg/mL respectively, p<0.05). Vitamin B_12_ levels showed no difference between those who did or did not consume meals, including meat in the last three days. However, comparison of levels in subjects who received meals, including meat in the last three days, showed that subjects consuming fish had higher levels than did subjects receiving chicken (290.9±146.9 pg/mL vs 242.4±115.0 pg/mL respectively, p<0.05). No significant differences existed among the groups in terms of the frequency of offal consumption.

Subjects consuming butter at breakfast had higher vitamin B_12_ levels than those who never ate breakfast (303.9±187.9 pg/mL vs 220.0±89.1 pg/mL respectively, p<0.05). Subjects consuming milk at breakfast had significantly higher vitamin B_12_ levels when compared with those drinking tea or nothing (297.5±145.1 pg/mL vs 252.6±136.8 pg/mL and 244.8±75.3 pg/mL respectively, p<0.05).

**Table 2.** Serum vitamin B_12_ levels (pg/mL) of subjects aged 1 month to 24 years

Age	Gender	Number	Median	P_10_- P_90_	P_5_- P_95_	P_2.5_- P_97.5_
1-12 month(s)	Male	17	210	143-400	140-418	140-418
	Female	21	243	134-560	122-647	121-655
1-5 year(s)	Male	112	264	146-493	133-588	110-681
	Female	100	268	175-526	132-625	125-686
6-11 years	Male	120	235	151-420	140-466	133-557
	Female	120	264	152-426	138-468	109-589
12-17 years	Male	120	200	142-378	130-397	116-570
	Female	120	215	141-381	133-451	128-615
18-24 years	Male	140	232	147-359	137-425	126-473
	Female	140	217	143-330	134-399	127-449
Total	Male	509	229	147-410	136-465	127-576
	Female	501	233	148-400	134-498	127-606

P=Percentile

**Table 3.** Serum vitamin B_12_ levels (pg/mL) in cord-blood and in newborns

Source of blood	Gender	Number	Median	P_10_-P_90_	P_5_-P_95_	P_2.5_-P_97.5_
Cord-blood	Male	28	181	127-613	121-740	119-1,060
	Female	26	170	148-300	147-1,089	147-1,500
	Total	54	174	147-417	126-793	121-1,335
Newborns	Male	23	181	132-429	119-700	116-532
	Female	22	206	137-750	127-1,178	126-1,230
	Total	45	194	135-446	127-775	121-1,178

P=Percentile

Vitamin B_12_ levels were significantly higher in infants [0-24 month(s), n=135] who were only on complementary feeding compared to those receiving only mother's milk (344.0±190.1 pg/mL vs 249.5±180.6 pg/mL respectively, p<0.05).

## DISCUSSION

The reference levels of vitamin B_12_ found in the current study were different from the levels found in studies conducted in other countries (Table 4). One of the characteristics of vitamin B_12_ is that its normal values differ between races and societies (4,11,12). This characteristic property makes it necessary to determine the acceptable normal values of vitamin B_12_ in each society. Carmel reported that the blacks have significantly higher cobalamin and transcobalamin (especially transcobalamin II) levels than whites do while the lowest levels were observed in India, Africa, and Pakistan (5). Recent reports show that vitamin B_12_ levels in people of European societies are substantially higher (13,14). However, the results from this study show significant differences among the groups investigated with respect to the number and age of subjects. Furthermore, the methodologies used in the studies of different countries vary widely. This situation precludes the possibility of making a suitable comparison. Ortega *et al*. pointed out similar observations while evaluating relavent studies conducted in Spain (13).

The dissemination of health services and increase in general knowledge among the population has served to increase the number of those benefiting from preventive health services. This has facilitated a decrease in the incidence of various diseases, such as avitaminosis A and D, which were previously prevalent. At the same time, vitamin B_12_ deficiency has attracted increased attention. Vitamin B_12_ deficiency was found in 11% of the Guatemalan school children (15). Vitamin B_12_ deficiency is also not rare in Turkey (16).

A review of studies conducted on vitamin B_12_ levels among children show different results as shown in Table 4. In the study of Davis *et al*., infants of 4-37 weeks had higher 95% CI limit and mean value and lower 5% limit and mean value of vitamin B_12_ when compared with our results (17). The values obtained in other studies (mean and percentage limit values), however, were higher than the values found in this study (8,13,18-24). These differences might be attributed to several factors, particularly the criteria and requirements for inclusion into the study. Davis *et al.* accepted only good health status and being breastfed as requirements for inclusion into the study (17). The vitamin B_12_ levels in the infants’ blood and their umbilical cord are closely related to the level in the mothers’ blood (16,25). However, the drugs containing vitamin B_12_ used during pregnancy and after giving birth and similar drugs that can be given to the infants have an increasing effect on vitamin B_12_ levels. Among other researchers, only Huemer *et al*. indicated the absence of any vitamin drug as a requirement for inclusion in their study (22). Thus, differences in the inclusion criteria for study subjects could affect the study results.

**Table 4.** Previous studies on vitamin B_12_ levels among children

Investigator, year	Country	Inclusion criteria	No.	Vitamin B_12_ levels	Results
Hages M, 1985	Germany		165	1-5 Y*: ^κ^591.7 (257-1,349) pg/mL 6-10 Y: 556.4 (234.4-1,349) pg/mL 11-15 Y: 468.1 (204.2-1,071.5) pg/mL	
Osifo BOA, 1986	Nigeria	Good health and, for girls, not during menstruation	240	12-17 Y (GP): ^η^615±258 (280-1,400) pmol/L 12-17 Y (Male): 554±202 (290-1,150) pmol/L 12-17 Y (Female): 687±298 (280-1,400) pmol/L	Higher in girls than boys, suggesting vitamin B_12_ levels in girls have some hormonal influences
Davis RE, 1986	Australia	Good health and receiving breastmilk	223	4-37 weeks: ^θ^334 (120-800) pg/mL	Levels were higher in infants being fed formula or cow's milk than fed breastmilk
Hicks JM, 1993	USA	Random	1,486	0-1 Y (Female): ^π^168-1,116 pmol/L 0-1 Y (Male): 216-891 pmol/L 13-18 Y (Female): 158-637 pmol/L 13-18 Y (Male): 134-605 pmol/L	
Ortega RM, 2001	Spain	76 studies reviewed	1,490	0-15 Y: ^α^679.7±127 pg/mL	In this country, daily vitamin B_12_ intake is 8-9 mcg while 0.9-2.2 mcg is recommended. Levels are low in 0-18% of the subjects
Shen M-H, 2002	Taiwan	Maintaining usual diet in the last 3 days	1,235	12-15 Y (Male): ^α^444.8±158.4 pg/mL 12-15 Y (Female): 495.0±181.3 pg/mL	Levels are lower in boys
Leoncini R, 2004	Mozambique	Healthy children on a standard diet	173	6-16 Y: ^α^782.7±537.1 pg/ml**	Level of Italians with similar age-group were 520±190 pg/mL
Huemer M, 2006	Austria	Good health and not receiving any vitamin or other drugs	264	2-5 Y: ^κ^572 (202-1,345) pg/mL 6-9 Y: 559 (201-1,050) pg/mL 10-13 Y: 437 (163-889) pg/mL 14-17 Y: 355 (142-736) pg/mL	No difference between genders but levels decreased with age
Obeid R, 2006	Germany	Children of healthy pregnant women over 17 years, premature and in-utero growth-retarded babies also included	92	Cord-blood: ^κ^268 (88-1,018) pmol/L	Cord-blood levels are higher than levels in mothers
McLean ED, 2007	Kenya	Randomly-chosen school children	120	6-14 Y: ^α^292±144 pmol/L	Nutrition with foods from animal source increases the levels

*Folic acid and cobalamin units might have been mixed in the text; GP=General population; Y=Years old; α=Mean±SD; η=Mean±SD (Minimum-Maximum); θ=Mean (P_5_-P_95_), κ=Mean (Minimum-Maximum) π=P_2.5_-P_97.5_; According to the international unit system, conversion coefficient pg/mL to pmol/L is 0.74’ (pmol/L=pg/mLx0.74)

Another reason for the differences observed between other studies and the current study can be nutritional habits. Disorders relating to excessive nutrition, such as obesity, are quite common in western societies; people in those societies are well-nourished, even excessively so. In Finland, subjects have been advised to take two mcg of vitamin B_12_ daily, yet their daily vitamin B_12_ intake was 7.4-11 mcg (12). The current study accepted consumption of red meat twice per week as an adequate nutritional sign of vitamin B_12_ intake. The findings regarding the nutritional habits of the participants indirectly confirmed that they had consumed red meat at least twice per week. If participants reported that they had consumed red meat at least twice per week despite they had actually consumed less, their vitamin B_12_ levels would be low. In that case, the vitamin B_12_ levels in participants who stated that they had consumed meat daily and preferred fish and food of animal origin at breakfast should have shown a significant difference when compared with those who did not prefer these types of food. However, examination of the results showed no significant difference. Meat consumed in this quantity, together with the consumption of other foods of animal origin, maintained an adequate vitamin B_12_ level but did not lead to a significant difference. This also shows that subjects who stated they consumed red meat at least twice per week were reporting accurately.

The following two points can help explain the low values observed in our study:

*(i) Ethnic diversity*: Some researchers have noted differences in vitamin B_12_ levels between the members of the white and black races (4,11,12). Kwee *et al*. reported vitamin B_12_ levels of 382±131.3 pg/mL and 546±197.5 pg/mL in healthy white and black females respectively. They stated that the difference between the two races was significant (4). A similar characteristic might affect other societies, and one might question whether ethnic characteristics have affected the results of the current study. It would be presumptuous to provide a definitive answer to this question in view of the inadequate number of studies on the issue. However, evidence exists that would lead us to think the opposite. In a study conducted in Australia, 56 of the participants were Turkish (22). In that study, while the vitamin B_12_ level of Turkish children was reported to be 592±70 pg/mL, that of Australian children was 469±79 pg/mL. This finding shows that Turkish people living in the same region with Australians and with similar opportunities for nutrition do not have low vitamin B_12_ levels.

*(ii) Vitamin B_12_ content of nutriments*: The source of vitamin B_12_ is food of animal origin. Animals do not produce this vitamin themselves. Animals eat food containing B_12_-producing bacteria; thus, the animals become sources of vitamin B_12_ (26). One explanation of low levels of vitamin B_12_ in humans could be low levels of cobalt in the soil, resulting in fewer microorganisms producing vitamin B_12_ by using cobalt in the region's soil. There is insufficient information to confirm or reject such a hypothesis.

Another reason for the different results among studies might be the differences between the methodologies used. The current study utilized the Beckman kits that are used in the biochemistry laboratory of the hospital for measuring vitamin levels. The reference range of 127-590 pg/mL obtained in the study was different from the reference range of the kits (180-914 pg/mL). The study by Christenson *et al.*, conducted on 154 healthy subjects, investigated the reference ranges using two different kits. Reference ranges were found to be 116-817 ng/L with the SimulTRAC-S kit and 205-810 ng/L with the Quantaphase kit (27). It is remarkable that there is a substantial difference between these results. The reference ranges of the kits were 180-960 and >200 ng/L respectively. Furthermore, false positivity was detected in 9% of the subjects with the first kit. Thus, Christenson *et al.* suggested that studies on normal range be completed in a society before assays are used in the evaluation of patients; the current authors support this idea (27). Kumar *et al.* compared three methods in a study conducted on the Indians, and they recommended radioisotope dilution assay as an accurate procedure for determining vitamin B_12_ levels (28).

In the current study, we found that when the factors that can affect vitamin B_12_ levels such as age, weight, height, and BMI increased, vitamin B_12_ levels were significantly lowered. In line with the current findings, others have reported that vitamin B_12_ levels significantly decrease with age (6,7,18,22,24,29). When subjects were stratified according to gender, it was found that vitamin B_12_ levels were significantly higher in females at the age of 7 and 14 years and in males at the age of 21 years. However, the findings about the association of gender and vitamin B_12_ levels are incompatible with the literature (6,8,11,18,20,22,29).

In this study, the serum level of vitamin B_12_ was not affected by the parents’ occupations or the levels of family income. This finding gave rise to the thought that people living in the region had good awareness of nutritional issues. The finding that vitamin B_12_ levels in children whose mothers had a university degree and whose fathers had high school education were significantly higher suggests education can be an effective factor in nutrition.

An interesting result of this research was that vitamin B_12_ levels were significantly higher in subjects who consumed fish when compared with those who consumed chicken. No comparable result could be found in the literature. This situation can be explained because the vitamin B_12_ level in fish is 10 times higher than in chicken (30). It is not surprising that infants taking complementary food had higher vitamin B_12_ levels compared to infants who were only breastfed. Infants are generally given food prepared with milk as a supplement. The vitamin B_12_ content in cow's milk is 5 to 10 times higher than that in human milk (30,31). The findings of the current study were supported by those of Davis *et al*. and Karademir *et al*. who reported that vitamin B_12_ levels in infants who were only breastfed were lower when compared with levels in infants who were fed with cow's milk and formula milk (17,32).

### Conclusions

The results of this research suggest that vitamin B_12_ levels vary among countries and that using reference ranges of varied populations may lead to inaccurate results. Therefore, the researchers advise that it would be beneficial to achieve normal levels that are valid for each population.

## ACKNOWLEDGEMENTS

We thank the Scientific Research Projects Coordination Office of Selcuk University for financial support. We also thank the medical doctors of the primary healthcare centres and other staff for their assistance in obtaining samples. Finally, we would like to thank Nazım Sonmez for analyzing the samples.
